# Effects of a games-based physical education lesson on cognitive function in adolescents

**DOI:** 10.3389/fpsyg.2023.1098861

**Published:** 2023-03-14

**Authors:** Luke M. Gilbert, Karah J. Dring, Ryan A. Williams, Ruth Boat, Caroline Sunderland, John G. Morris, Mary E. Nevill, Simon B. Cooper

**Affiliations:** Sport, Health, and Performance Enhancement (SHAPE) Research Centre, Department of Sport Science, School of Science and Technology, Nottingham Trent University, Nottingham, United Kingdom

**Keywords:** physical education, physical activity, cognition, intensity, fitness

## Abstract

Despite the importance of physical education (PE) lessons for physical activity in adolescents, the acute cognitive responses to PE lessons have not been explored; a gap in the literature that this study addresses. Following familiarisation, 76 (39 female) adolescents (12.2 ± 0.4 y) completed two trials (60 min games-based PE lesson and 60 min academic lesson) separated by 7-d in a counterbalanced, crossover design. Attention, executive function, working memory, and perception were assessed 30 min before, immediately post, and 45 min post-lesson in both trials. Participants were split into high-and low-fit groups based on a gender-specific median split of distance run on the multi-stage fitness test. Furthermore, participants were split into high and low MVPA groups based on a gender-specific median split of MVPA time (time spent >64% HR max) during the PE lesson. Overall, a 60 min games-based PE lesson had no effect on perception, working memory, attention, or executive function in adolescents (all *p* > 0.05) unless MVPA time is high. The physical activity-cognition relationship was moderated by MVPA, as working memory improved post-PE lesson in adolescents who completed more MVPA during their PE lesson (time*trial*MVPA interaction, *p* < 0.05, partial η^2^ = 0.119). Furthermore, high-fit adolescents displayed superior cognitive function than their low-fit counterparts, across all domains of cognitive function (main effect of fitness, all *p* < 0.05, partial η^2^ 0.014–0.121). This study provides novel evidence that MVPA time moderates the cognitive response to a games-based PE lesson; and emphasises that higher levels of fitness are beneficial for cognitive function in adolescents.

## Introduction

Chief Medical Officer guidance states that young people aged 5–18 years should participate in an average of 60 min per day moderate-to-vigorous physical activity (MVPA) to enhance health and well-being (UK Chief Medical Officers, 2019); yet recent data suggest that only 44.6% of children and adolescents in England achieve these recommendations (Sport England, 2021). Given the large proportion of time that young people spend in school, schools present a unique opportunity to assist young people in meeting the daily physical activity recommendations (Hills et al., 2015). Consequently, schools are often designated as a promising environment for increasing physical activity in *all* young people, irrespective of their background (Slingerland et al., 2012; van Slujis et al., 2021). Within the school environment, as it is compulsory in most Western school systems, physical education (PE) lessons have been identified as an important source of physical activity (Fairclough and Stratton, 2005) and provide adolescents with the opportunity to achieve the recommended 60 min of MVPA per day (Hills et al., 2015). Physical activity opportunities provided by PE lessons are of particular significance for the least active students (Fairclough and Stratton, 2005), with evidence that 30% of adolescents from Western Europe derive all of their daily MVPA from PE lessons (Westerstahl et al., 2005); suggesting that PE lessons are indeed the only form of MVPA for some adolescents (Association for Physical Education, 2020).

Despite the significance of PE as an opportunity for adolescents to participate in MVPA (Zhou and Wang, 2019), the time allocated for PE (and physical activity more broadly) in the school curriculum has often been reduced to accommodate increased instructional time for academic subjects (Rasberry et al., 2011; van den Berg et al., 2016). In the UK for example, the prioritisation of academic subjects is illustrated by a 13% reduction in the number of taught hours for PE since 2011/12; during which time mathematics, English, and science have increased by 13, 11, and 14%, respectively (National Statistics, 2021). However, the reduction in teaching hours for PE is somewhat counterintuitive given that physical activity is positively associated with cognition and academic achievement (Sneck et al., 2019; Garcia-Hermoso et al., 2021). Furthermore, evidence suggests that increasing PE time does not negatively impact academic achievement, even when less time is dedicated to subjects other than PE (Rasberry et al., 2011); thus, reducing PE time (and subsequently physical activity) could be counter-productive for enhancing academic achievement and cognitive function.

Cognitive function can be defined as a variety of brain-mediated functions and processes (Schmitt et al., 2005). These functions allow us to perceive, evaluate, store, manipulate, and use information from external (e.g., environment) and internal (e.g., experiences, memory) sources, before responding to this information (Schmitt et al., 2005). Cognitive functions are clustered into the six domains of executive function, memory, attention, perception, language, and psychomotor functions (Schmitt et al., 2005). A recent meta-analysis by Haverkamp et al. (2020) concluded that acute exercise interventions improved cognitive outcomes (Hedges’ *g* = 0.31). Specifically, processing speed (*g* = 0.39), attention (*g* = 0.34) and inhibition (a component of executive function; *g* = 0.32), were enhanced in adolescents following physical activity. This is of importance given that these cognitive domains are the foundation of academic ability (Esteban-Cornejo et al., 2015), Furthermore, as adolescence is a critical stage for the development of cognitive function (Romeo and McEwen, 2006), and a period during which cognitive function and academic achievement are a key focus of the education sector, physical activity opportunities in schools are of particular importance for this population.

The positive effects of physical activity on cognitive function are influenced by several factors such as physical fitness (Garcia-Hermoso et al., 2021), the characteristics of the physical activity (intensity, duration, and modality) and the domain of cognitive function assessed (Williams et al., 2019). Despite the influence of these moderating factors, running is the most common exercise modality in research examining the acute effects of physical activity on adolescent cognitive function. Whilst running appears to be an effective modality (e.g., Budde et al., 2010; Cooper et al., 2012; 2016), it does not reflect typical activity patterns in adolescents (Rowlands et al., 2008) and does not foster long-term adherence (Howe et al., 2010). Recent research has attempted to replicate the high-intensity and intermittent activity patterns that are preferred by adolescents (Bailey et al., 1995) and has demonstrated the positive acute effects of games-based activity (such as basketball) on subsequent cognitive function (Cooper et al., 2018). The positive influence of games-based activity, an activity that requires cognitive engagement, is consistent with the belief that cognitively engaging physical activities elicit a greater effect on subsequent cognitive function (Crova et al., 2014). However, previous studies that have examined games-based activities have done so through direct intervention of researchers and the provision of a games-based activity that was not delivered within the PE curriculum. The applicability of such findings to curriculum delivered PE lessons is thus unknown. Therefore, developing an understanding of the activity patterns of PE, and how PE influences subsequent cognitive function, is an important gap in existing research that should be addressed.

Previous research investigating PE in adolescents has primarily focussed on the activity volume, intensity, and patterns (Fairclough and Stratton, 2005; Slingerland et al., 2011; Hollis et al., 2017; Lyyra, et al., 2017; Mooses et al., 2017; Cheung, 2019; Zhou and Wang, 2019; Wallace et al., 2020); with no examination of the acute cognitive response to a single PE lesson in adolescents. Whilst a recent systematic review and meta-analysis concluded that there were no statistically significant effects of secondary PE interventions on cognitive function in adolescents (i.e., aged >11 years; Garcia-Hermoso et al., 2021), the review was only able to analyse the effect of chronic (> 12 weeks) PE interventions on adolescent cognitive function due to a paucity of research that has examined the acute cognitive responses to a single bout of PE.

To date, the only study to investigate the acute cognitive response to a single PE lesson was conducted in primary school-aged children (aged 8–9 years) and reported no effect of a single PE lesson on memory and attention, when compared to no physical activity (Pirrie and Lodewyk, 2012). However, as the PE lesson, through various activities, required students to undertake 20 min of MVPA by moving around the room performing specific movements (e.g., hopping), it was not reflective of the national curriculum for PE (Department for Education, 2013) or a typical PE lesson. Additionally, cognitive function testing was not administered simultaneously for all participants, with testing ranging from 10 min to 60 min post lesson. The timing of post-exercise cognitive function testing is an important consideration given that this timing has been shown to moderate the subsequent effects on cognition (e.g., Cooper et al., 2018; Hatch et al., 2021). Therefore, the lack of control of this key variable in previous work limits the conclusions that can be drawn regarding the acute effects of a PE lesson on subsequent cognitive function.

Therefore, the primary aim of the study was to examine the acute effects of a curriculum-based PE lesson on subsequent cognitive function in adolescents. A secondary aim was to quantify the physical activity characteristics of a game-based PE lesson, given that these activity patterns are likely to influence the subsequent effects on cognition. Finally, the third aim was to analyse whether there was a moderating effect of physical fitness, or the amount of MVPA completed during the lesson, on subsequent cognitive performance.

## Materials and methods

### Participant characteristics

To estimate our sample size, an *a priori* power analysis was conducted using G*Power version 3.1.9.7 (Faul et al., 2007). For a repeated measures approach (two groups, three measurements; two-tailed test; ⍺ = 0.05, power = 0.80), and a small effect size of 0.14 [based on the previous work by Cooper et al. (2018)], the minimum sample size was n = 84. Subsequently, eighty-five young people (aged 12–13 years) were recruited to participate in the study. However, nine participants failed to complete the study due to absence from school for one of the experimental trials. Therefore, a total of 76 participants completed the study (37 male, 39 female). During familiarisation, all participants underwent anthropometric measures of height, body mass, sitting height, waist circumference and skinfold thickness. A Leicester Height Measure (Seca, Germany), accurate to 0.1 cm, was used to measure height and a Seca 770 digital scale (Seca, Germany), accurate to 0.1 kg, was used to measure body mass. An estimation of maturity offset was made by measuring sitting height, to subsequently estimate years from peak height velocity using methods previously described (Moore et al., 2015). Body mass index (BMI) was calculated and subsequently age-and gender-specific centiles for BMI were derived based on national reference values (Cole et al., 2000). Four skinfold sites were measured (triceps, subscapular, supraspinale, and front thigh) using previously described methods, as a marker of body composition (Dring et al., 2018). Participants were split into high-fit and low-fit groups based on a gender-specific median split of distance run on the multi-stage fitness test (as per previous research (Cooper et al., 2018; Williams et al., 2020)). Likewise, participants were split into high MVPA and low MVPA groups based on the gender-specific median split on MVPA time during the PE lesson. Descriptive participant characteristics are presented in Table 1.

**Table 1:** Participant characteristics for the group overall, as well as for the high-and low-fit groups, and high and low MVPA groups.

**Variable**	**Overall**	**High-fit (n = 38)**	**Low-fit (n = 38)**	***p* value** ^a^	**High-MVPA (n = 35)**	**Low-MVPA (n = 36)**	***p* value** ^b^
Age (y)	12.2 ± 0.4	12.2 ± 0.4	12.2 ± 0.4	0.695	12.2 ± 0.4	12.2 ± 0.4	0.959
Height (cm)	157.3 ± 8.1	157.9 ± 7.7	156.7 ± 8.5	0.547	156.6 ± 7.4	158.0 ± 9.1	0.484
Body mass (kg)	49.0 ± 10.3	46.3 ± 7.1	51.6 ± 12.2	0.025*	47.6 ± 9.7	51.2 ± 11.1	0.159
Body mass index (BMI; kg^.^m^2^)	19.7 ± 3.4	18.5 ± 1.8	20.9 ± 4.0	0.002*	19.3 ± 3.1	20.4 ± 3.6	0.175
BMI percentile	64.5 ± 25.7	56.9 ± 22.0	71.6 ± 27.1	0.012*	61.2 ± 28.1	70.7 ± 22.5	0.120
Waist circumference (cm)	66.8 ± 8.1	63.6 ± 4.6	69.7 ± 9.6	< 0.001*	66.1 ± 8.0	68.4 ± 8.2	0.242
Sum of 4 skinfolds (mm)	64.2 ± 24.7	54.5 ± 16.4	73.4 ± 27.8	< 0.001*	61.5 ± 23.2	68.1 ± 27.1	0.273
Maturity offset ^c^	−0.30 ± 0.91	−0.26 ± 0.89	−0.36 ± 0.93	0.645	−0.44 ± 0.93	−0.20 ± 0.86	0.245
MSFT distance (m)	880 ± 320	1,100 ± 260	660 ± 180	< 0.001*	900 ± 300	860 ± 340	0.655

Abbreviations: MSFT Multi-Stage Fitness Test

a comparison between high and low fit, independent samples *t* -test.

b comparison between high and low MVPA during the PE trial, independent samples *t* -test.

c calculated using the method of Moore et al. (2015).

* significant difference (*p* < 0.05) between high and low fit groups.

### Study design

Following approval from the institution’s ethical advisory committee (approval number SST-659), participants were recruited from a secondary school in the East Midlands, UK. As per the guidelines for school-based research, head teacher consent was gained. Additionally, written informed consent from parents/guardians and a health screen questionnaire were completed for each participant; this determined each participant’s eligibility for participation by screening for health conditions which may be affected by participation (e.g., exercise-induced asthma). Participants also provided their written assent to participate in the study.

The study employed a randomised, order-balanced, crossover, within-subjects design, consisting of two main experimental trials (PE lesson and academic lesson). A familiarisation took place ~7 d before the first main trial, whereby the protocol of the study was explained to the participants, and they were provided with the opportunity to practice and become familiar with the procedures to be used, including the cognitive function tests. The procedures of the study were also provided to parents/guardians before the study *via* both written information and a phone call from a member of the research team. Opportunities were provided for participants/parents/guardians to ask questions to clarify any aspect of the study they did not fully understand.

To assess physical fitness participants completed the multi-stage fitness test (MSFT; Ramsbottom et al., 1988) during the familiarisation trial. The MSFT involves progressive 20 m shuttle runs in time with an audio signal, until volitional exhaustion or the point at which participants could not maintain the required running speed to keep time with the audio signal. The MSFT commenced at a speed of 8.0 km^.^h^−1^, increased by 1.0 km^.^h^−1^ to 9.0 km^.^h^−1^ for stage two, and increased by 0.5 km^.^h^−1^ for every completed stage thereafter. To monitor heart rate throughout the MSFT (and record maximum heart rate upon completion), participants were fitted with a chest-worn heart rate monitor (Firstbeat Team Sport System; Firstbeat Technologies Ltd., Finland). To encourage maximum effort from the participants, the research team provided verbal encouragement and participants were paced by an experienced member of the research team familiar with the test. Performance on the MSFT was determined by the total distance covered (m). Using a gender-specific median split of distance ran on the MSFT, participants were split into high-fit and low-fit groups [as per previous research (Cooper et al., 2018; Williams et al., 2020)].

**Figure 1:**
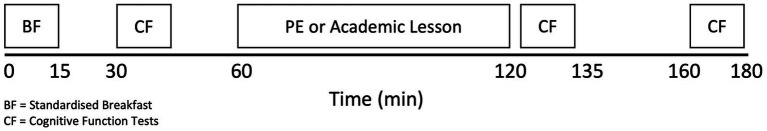
Experimental protocol.

As outlined in Figure 1, 60 min following breakfast, participants attended either a 60 min PE lesson or a 60 min academic lesson. A battery of cognitive functions tests were completed 30-min pre-, immediately post-and 45-min post-each lesson.

### Pre-trial control

The evening before their first experimental trial, participants consumed a meal of their choice and repeated this for their subsequent experimental trial. Subsequently, participants were asked to fast from 10 pm the evening before each experimental trial. To maintain euhydration, water was allowed *ad libitum* during this time. Additionally, for 24 h prior to each experimental trial participants were also asked to avoid any unusually vigorous physical activity. Parents/guardians were contacted by telephone the evening prior to each experimental trial to ensure compliance with these requirements. All participants followed the pre-trial requirements. On the morning of each experimental trial, participants reported to the school (between 8:45 am and 8:55 am) and consumed a standardised breakfast consisting of cornflakes, milk, and toast; providing 1.5 g carbohydrate per kg of body mass, identical to the breakfast of Williams et al. (2020). A standardised breakfast was provided to control for the potential of breakfast and exercise to interact and affect cognitive function in young people (Cooper et al., 2015).

### Lesson protocol

The single-gender PE lessons consisted of a 60 min football session, completed outdoor on a rubber crumb pitch. Football was selected as the activity given its popularity among young people and within the PE curriculum. A single researcher was present during the PE lesson to facilitate heart rate and GPS data collection and to provide a description of the lesson. The single researcher present played no active part in the lesson, and they did not interact with the participants or teacher. The PE lessons consisted of a warm-up, skill-based drills, and small-sided games. All lessons were delivered by the participants’ normal PE teacher and the research team did not influence the nature or focus of the session. Throughout both experimental trials, participants were fitted with a heart rate monitor (Firstbeat Team Sport System; Firstbeat Technologies Ltd., Finland). Heart rate was monitored continuously throughout both trials. Maximum heart rate and average heart rate were recorded for each trial. Participants were removed from analyses where heart rate data was incomplete or missing (n = 5), thus 71 participants were included for heart rate analysis. For the PE trial only, participants were also fitted with a PlayerTek Global Position System (GPS) unit (Catapult Sports, Melbourne, Australia). The units were placed outside and left stationary to enable an accurate number of satellite signals to be obtained (> 6 satellites). Once satellite signals were obtained, units were placed between the scapulae using an elasticated shoulder harness. The mean satellite signal strength was 9 ± 1 and horizontal dilution of precision was 1.00 ± 0.16. Participants were removed from analyses where GPS data was incomplete or missing (n = 13), thus 63 participants were included for GPS analysis. Variables of interest were total distance covered (m) and distance covered at low (<9 km^.^h^−1^), moderate (9–13 km^.^h^−1^), and high-speed (>13 km^.^h^−1^) (based on the speed zones of previous research; Randers et al., 2014). MVPA time was calculated as the percentage of the timetabled lesson time spent above 64% HR max, in accordance with ACSM guidelines (American College of Sports Medicine, 2017). For the academic lesson, participants attended their timetabled 60 min lesson in mathematics (n = 32), geography (n = 16), philosophy and ethics (n = 13), or personal development (n = 14); as per their normal school timetable. As outlined in Figure 1, both trials followed a time-matched protocol, with the only difference being the lesson attended (i.e., PE or academic).

### Cognitive function tests

The battery of cognitive function tests lasted approximately 12 min and consisted of the Stroop test, Sternberg paradigm, and visual search task; completed in that order on a laptop computer (Lenovo ThinkPad T450; Lenovo, Hong Kong). Preceding each cognitive function test and level, the instruction was presented on the screen to each participant and participants completed 3–6 practice stimuli to re-familiarise with the test, negating any potential learning effects; data for these practice stimuli were discarded. The battery of cognitive function tests were completed in silence, in a classroom, and participants were separated such that they could not interact during the tests. To minimise external disturbances, participants wore sound cancelling headphones and the room lights were dimmed to enhance screen visibility. For all cognitive function tests, participants were instructed to respond as quickly and accurately as possible. This testing procedure has been previously used successfully in a similar study population (e.g., Cooper et al., 2018; Williams et al., 2020). For each cognitive function test, the variables of interest were response time (ms) of correct responses and the proportion (%) of correct responses made. To prevent the influence of unusually slow or fast responses on the analyses, response times were filtered in accordance with procedures previously conducted (Cooper et al., 2018), with minimum (< 100 ms) and maximum (2,000–10,000 ms, depending on task complexity) response time cut-offs applied.

### Stroop test

To measure selective attention and executive function, the Stroop test was administered (Miyake et al., 2000). The Stroop test consists of two levels, simple and complex. Both levels of the Stroop test involved a test word being presented in the centre of the laptop screen, with a target and a distractor randomly presented on the left and right sides of the screen. Using the appropriate arrow key (left or right), participants were instructed to select their responses. For the simple level, the test word, target word and distractor word were all presented in a white font; a total of 20 stimuli were presented. On the complex level (colour-interference) there were 40 stimuli, with the participant selecting the colour the word was written in rather than the word itself (e.g., if ‘green’ was written in blue font, the correct response would be blue). Choices remained on the screen until the participant responded, with an inter-stimulus interval of 1 s.

### Sternberg paradigm

The Sternberg paradigm is a commonly used test that measures the domain of working memory (Sternberg, 1969). The test consists of three levels of ascending complexity that utilise a different working memory load (one, three or five items). The one item level consisted of 16 test stimuli and the number ‘3’ is always the target. Whereas on the three and five item levels, the target is three (e.g., ‘A F P’) or five (e.g., ‘B E H R V’) randomly generated letters, respectively; with each containing 32 test stimuli. At the start of each level, the target items were displayed along with instructions to press the right arrow key if a target item was presented and the left arrow key otherwise. The correct response was counterbalanced between the left and right arrow keys for each level. On all levels, the choice stimuli were presented in the centre of the screen, with an inter-stimulus interval of 1 s.

### Visual search

The visual search test comprised two levels; simple and complex. When completing both levels of the visual search test, participants were instructed to press the space key as soon as they could detect a triangle on the screen. Following each response, a new target would appear following a random delay (minimum 500 ms delay). The simple level assessed simple visuomotor speed and required participants respond to 20 targets, which were triangles drawn in solid green lines on a black background. For the complex level, participants responded to 40 targets. The additional complex visual processing component of a background distractor was introduced, induced by random moving dots on the screen (to induce the distracting visual effect of a flickering background, a new set of distractor dots were re-drawn on the screen every 250 ms). Target triangles were initially drawn with just a few visible dots of each line, and the density of these points increased linearly with time until the participants responded.

## Statistical analysis

Response time and accuracy analyses for the cognitive function tests were conducted using R (www.r-project.org). Analyses were conducted using a two-way (trial*time) repeated measures analysis of variance (ANOVA) for each test level, as each level requires a different level of cognitive processing. Prior to analyses, response times were log transformed to exhibit the right-hand skew, typical of human response times. To assess the moderating effect of fitness and MVPA on the exercise-cognition relationship, three-way (trial*time*fitness and trial*time*MVPA) repeated measures analysis of variance (ANOVA) were conducted for each variable from the cognitive function tests. Collinearity between fitness and MVPA time was assessed using Spearman’s rank-order correlation. Where statistically significant three-way interactions existed, post-hoc two-way (trial * time) ANOVA were conducted separately for high-and low-fit adolescents (for trial * time * fitness interactions) and high-and low-MVPA adolescents (for trial * time * MVPA interactions). For all statistically significant effects, partial eta squared effect sizes are included and interpreted as per convention (i.e., 0.01: small; 0.06: medium; 0.14: large).

Maximum heart rate, average heart rate, total distance covered, and distance covered at low, moderate, and high speed during the PE lesson were compared between groups (high-*vs* low-fit; high-*vs* low-MVPA) using SPSS (version 28; SPSS Inc., Chicago, IL., USA) using independent samples t-tests. All data are presented as mean ± standard error of the mean (SEM), unless stated otherwise. Statistical significance was accepted as *p* < 0.05.

## Results

### Lesson characteristics

Descriptive data for the PE lessons are presented in Table 2. During the PE lessons, time spent in MVPA (*p* = 0.445), average heart rate (*p* = 0.093), total distance covered (*p* = 0.094), and the distance covered at low (<9 km^.^h^−1^; *p* = 0.181), moderate (9–13 km^.^h^−1^; *p* = 0.096), and high (>13 km^.^hr.^−1^; *p* = 0.200) speeds were similar between the high and low fit adolescents. However, the maximum heart rate during the PE lessons was significantly higher for the high fit adolescents when compared to low fit adolescents (high-fit; 200 ± 8 beats^.^min^−1^, low fit; 195 ± 11 beats^.^min^−1^; *t*_(69)_ = 0.768, *p* = 0.046, *d* = 0.48). Furthermore, during the PE lesson, total distance (*p* = 0.807), and the distance covered at low (*p* = 0.398), moderate (*p* = 0.884), and high (*p* = 0.290) speeds were similar between the high and low MVPA adolescents. There was no relationship between fitness and MVPA time during the PE lesson (*r_s_* = 0.130, *p* = 0.281).

**Table 2:** Descriptive data and inferential statistics for the PE lessons overall, as well as for the high fit and low fit groups, and the high MVPA and low MVPA groups.

**Variable**	**Overall**	**High-fit**	**Low-fit**	***p* value** ^a^	**High-MVPA**	**Low-MVPA**	***p* value** ^b^
MVPA [% total time]	67 ± 14	68 ± 15	65 ± 13	0.445	78 ± 5	56 ± 11	
Average heart rate [beats^.^min^−1^]	149 ± 12	151 ± 12	146 ± 12	0.093	157 ± 8	140 ± 9	< 0.001**
(% HR max)	72 ± 6	73 ± 6	70 ± 6		75 ± 4	67 ± 4	
Maximum heart rate [beats^.^min^−1^]	197 ± 10	200 ± 8	195 ± 11	0.046*	204 ± 7	191 ± 9	< 0.001**
(% HR max)	95 ± 5	96 ± 4	94 ± 5		98 ± 3	92 ± 4	
Total distance [km]	2.49 ± 0.46	2.58 ± 0.44	2.39 ± 0.47	0.094	2.47 ± 0.47	2.50 ± 0.48	0.807
Distance at low speed [km]	1.88 ± 0.26	1.92 ± 0.21	1.84 ± 0.29	0.181	1.85 ± 0.28	1.90 ± 0.25	0.398
Distance at moderate speed [km]	0.41 ± 0.16	0.44 ± 0.17	0.37 ± 0.16	0.096	0.41 ± 0.16	0.41 ± 0.18	0.884
Distance at high speed [km]	0.20 ± 0.12	0.22 ± 0.13	0.18 ± 0.11	0.200	0.21 ± 0.12	0.18 ± 0.13	0.290

Data presented as mean ± SD.

a comparison between high and low fit, independent samples t-test.

b comparison between high and low MVPA during the PE trial, independent samples t-test.

* significant difference (*p* < 0.05) between high and low fit groups.

** significant difference (*p* < 0.05) between high and low MVPA groups.

### Cognitive function tests

Data for each of the cognitive function tests, across both trials, are displayed in Table 3 (overall), Table 4 (split by fitness group) and Table 5 (split by MVPA group).

**Table 3:** Cognitive function data across the academic and PE trials.

Test	Level	Variable	Academic trial			PE trial		
			Pre-exercise	Immediately post-exercise	45 min post-exercise	Pre-exercise	Immediately post-exercise	45 min post-exercise
Stroop test	Simple	Response time [ms]	811 ± 20	766 ± 18	773 ± 20	800 ± 17	770 ± 18	768 ± 18
		Accuracy [%]	97.6 ± 0.5	95.7 ± 0.7	94.6 ± 0.9	97.4 ± 0.4	95.7 ± 0.7	96.2 ± 0.6
	Complex	Response time [ms]	1,103 ± 33	1,032 ± 30	1,037 ± 29	1,111 ± 26	1,068 ± 24	1,065 ± 25
		Accuracy [%]	93.0 ± 1.2	92.2 ± 1.0	92.3 ± 1.0	94.1 ± 0.7	93.3 ± 0.6	93.3 ± 0.7
Sternberg paradigm	One item	Response time [ms]	560 ± 15	510 ± 10	525 ± 13	549 ± 13	529 ± 13	533 ± 12
		Accuracy [%]	92.3 ± 1.6	93.3 ± 0.9	93.4 ± 0.8	95.1 ± 0.7	94.8 ± 0.7	94.8 ± 0.7
	Three item	Response time [ms]	674 ± 15	661 ± 14	663 ± 14	697 ± 14	681 ± 15	664 ± 14
		Accuracy [%]	93.5 ± 0.9	93.4 ± 0.6	92.7 ± 0.8	93.2 ± 1.3	93.1 ± 0.7	93.4 ± 0.9
	Five item	Response time [ms]	847 ± 19	796 ± 19	771 ± 19	840 ± 19	779 ± 17	793 ± 17
		Accuracy [%]	92.0 ± 0.7	88.0 ± 1.3	87.5 ± 1.4	90.2 ± 1.2	89.3 ± 1.2	86.9 ± 1.3
Visual search	Simple	Response time [ms]	591 ± 8	585 ± 6	590 ± 7	587 ± 8	594 ± 8	592 ± 8
		Accuracy [%]	89.4 ± 2.3	91.3 ± 1.7	89.8 ± 2.0	92.6 ± 2.1	93.3 ± 1.9	90.3 ± 2.4
	Complex	Response time [ms]	1,633 ± 46	1,580 ± 43	1,551 ± 42	1711 ± 38	1,574 ± 40	1,593 ± 44
		Accuracy [%]	92.4 ± 1.3	91.9 ± 1.4	92.6 ± 1.0	94.5 ± 1.2	92.7 ± 1.8	91.0 ± 2.1

Data are mean ± SEM.

**Table 4:** Cognitive function data across the academic and PE trials for the high and low fitness groups.

Test	Level	Variable	Participant Group	Academic trial			PE trial		
				Pre-exercise	Immediately post-exercise	45 min post-exercise	Pre-exercise	Immediately post-exercise	45 min post-exercise
Stroop test	Simple	Response time [ms]	Low Fitness	840 ± 32	812 ± 30	834 ± 33	830 ± 29	805 ± 29	807 ± 30
			High Fitness	781 ± 23	721 ± 18	713 ± 19	770 ± 16	735 ± 21	729 ± 16
		Accuracy [%]	Low Fitness	97.6 ± 0.6	96.3 ± 0.9	95.0 ± 1.0	97.5 ± 0.5	95.0 ± 1.1	95.6 ± 0.8
			High Fitness	97.5 ± 0.7	95.0 ± 1.1	94.2 ± 1.4	97.2 ± 0.5	96.3 ± 0.7	96.6 ± 0.7
	Complex	Response time [ms]	Low Fitness	1,167 ± 48	1,095 ± 45	1,123 ± 44	1,160 ± 44	1,107 ± 39	1,126 ± 40
			High Fitness	1,039 ± 43	968 ± 37	951 ± 32	1,063 ± 24	1,030 ± 28	1,004 ± 28
		Accuracy [%]	Low Fitness	91.3 ± 2.0	91.1 ± 1.8	91.5 ± 1.5	93.5 ± 1.3	92.4 ± 0.8	92.9 ± 1.1
			High Fitness	94.6 ± 1.0	93.2 ± 0.8	93.1 ± 1.1	94.6 ± 0.6	94.1 ± 0.8	93.6 ± 0.7
Sternberg paradigm	One item	Response time [ms]	Low Fitness	586 ± 24	535 ± 15	574 ± 22	581 ± 21	540 ± 21	563 ± 20
			High Fitness	534 ± 18	483 ± 11	473 ± 10	515 ± 12	518 ± 15	502 ± 11
		Accuracy [%]	Low Fitness	91.2 ± 1.7	93.4 ± 1.2	92.1 ± 1.3	94.4 ± 1.1	94.4 ± 1.1	95.2 ± 0.9
			High Fitness	93.4 ± 2.6	93.1 ± 1.4	94.8 ± 1.1	96.0 ± 0.8	95.2 ± 0.8	94.4 ± 1.0
	Three item	Response time [ms]	Low Fitness	715 ± 21	692 ± 22	701 ± 23	727 ± 23	706 ± 21	689 ± 19
			High Fitness	630 ± 20	628 ± 16	623 ± 13	665 ± 14	654 ± 19	638 ± 18
		Accuracy [%]	Low Fitness	94.1 ± 0.9	93.7 ± 0.8	93.4 ± 1.1	92.1 ± 2.5	92.4 ± 1.1	92.5 ± 1.5
			High Fitness	93.0 ± 1.5	93.2 ± 0.9	91.9 ± 1.3	94.3 ± 0.8	93.8 ± 0.9	94.3 ± 0.9
	Five item	Response time [ms]	Low Fitness	863 ± 23	817 ± 28	786 ± 30	871 ± 31	796 ± 27	776 ± 26
			High Fitness	830 ± 31	774 ± 24	755 ± 22	808 ± 22	761 ± 21	810 ± 22
		Accuracy [%]	Low Fitness	91.4 ± 1.0	87.3 ± 1.7	86.0 ± 2.0	88.5 ± 2.2	88.1 ± 1.8	85.3 ± 2.2
			High Fitness	92.6 ± 0.8	88.6 ± 1.9	89.2 ± 1.9	91.9 ± 1.1	90.5 ± 1.5	88.6 ± 1.5
Visual search	Simple	Response time [ms]	Low Fitness	605 ± 11	596 ± 10	587 ± 11	600 ± 12	601 ± 12	603 ± 10
			High Fitness	576 ± 10	574 ± 7	593 ± 8	573 ± 10	586 ± 11	580 ± 12
		Accuracy [%]	Low Fitness	87.3 ± 3.9	90.6 ± 2.8	91.0 ± 1.8	92.0 ± 3.2	93.6 ± 2.5	89.1 ± 3.5
			High Fitness	91.6 ± 2.2	92.0 ± 2.0	88.5 ± 3.7	93.2 ± 2.6	93.1 ± 2.9	91.4 ± 3.3
	Complex	Response time [ms]	Low Fitness	1,620 ± 62	1,579 ± 64	1,551 ± 62	1758 ± 51	1,586 ± 62	1,656 ± 70
			High Fitness	1,647 ± 70	1,580 ± 59	1,551 ± 56	1,661 ± 56	1,562 ± 50	1,525 ± 51
		Accuracy [%]	Low Fitness	91.8 ± 2.1	92.3 ± 1.9	92.7 ± 1.3	95.4 ± 1.6	90.1 ± 3.1	88.0 ± 3.5
			High Fitness	93.1 ± 1.6	91.4 ± 2.1	92.5 ± 1.6	93.6 ± 1.8	95.4 ± 1.5	94.2 ± 2.0

Data are mean ± SEM.

**Table 5:** Cognitive function data across the academic and PE trials for the high and low MVPA groups.

Test	Level	Variable	Participant Group	Academic trial			PE trial		
				Pre-exercise	Immediately post-exercise	45 min post-exercise	Pre-exercise	Immediately post-exercise	45 min post-exercise
Stroop test	Simple	Response time [ms]	Low MVPA	790 ± 23	746 ± 23	748 ± 27	775 ± 22	742 ± 20	744 ± 18
			High MVPA	834 ± 35	782 ± 30	795 ± 32	810 ± 28	785 ± 32	778 ± 31
		Accuracy [%]	Low MVPA	97.9 ± 0.6	96.5 ± 0.9	93.9 ± 1.0	96.7 ± 0.6	95.6 ± 1.1	95.4 ± 0.9
			High MVPA	97.1 ± 0.9	94.3 ± 1.2	95.0 ± 1.6	97.7 ± 0.5	95.6 ± 1.0	96.6 ± 0.8
	Complex	Response time [ms]	Low MVPA	1,066 ± 38	994 ± 41	992 ± 36	1,064 ± 30	1,022 ± 25	1,027 ± 34
			High MVPA	1,138 ± 57	1,086 ± 47	1,075 ± 49	1,143 ± 42	1,097 ± 44	1,095 ± 40
		Accuracy [%]	Low MVPA	94.5 ± 1.0	93.2 ± 1.0	93.4 ± 0.8	94.9 ± 0.6	93.3 ± 0.8	93.8 ± 0.7
			High MVPA	90.5 ± 2.3	91.2 ± 1.9	90.6 ± 1.9	92.9 ± 1.4	93.1 ± 1.0	92.3 ± 1.2
Sternberg paradigm	One item	Response time [ms]	Low MVPA	543 ± 21	503 ± 13	506 ± 17	517 ± 13	505 ± 14	518 ± 15
			High MVPA	591 ± 28	512 ± 17	539 ± 21	567 ± 22	539 ± 18	543 ± 18
		Accuracy [%]	Low MVPA	89.7 ± 3.0	93.4 ± 1.2	93.9 ± 1.3	94.9 ± 1.1	94.3 ± 1.1	94.8 ± 0.9
			High MVPA	94.1 ± 1.2	92.3 ± 1.7	92.3 ± 1.2	95.7 ± 0.9	95.2 ± 1.0	94.3 ± 1.0
	Three item	Response time [ms]	Low MVPA	655 ± 22	673 ± 22	644 ± 16	673 ± 18	669 ± 21	662 ± 20
			High MVPA	688 ± 22	658 ± 22	676 ± 22	713 ± 22	684 ± 19	663 ± 20
		Accuracy [%]	Low MVPA	93.4 ± 1.4	94.1 ± 0.8	93.5 ± 1.0	94.1 ± 1.0	92.1 ± 1.2	94.0 ± 0.8
			High MVPA	93.3 ± 1.1	92.5 ± 1.0	91.3 ± 1.4	91.6 ± 2.7	93.2 ± 0.9	92.1 ± 1.7
	Five item	Response time [ms]	Low MVPA	842 ± 29	800 ± 27	773 ± 26	797 ± 23	766 ± 24	792 ± 24
			High MVPA	858 ± 29	793 ± 28	762 ± 30	874 ± 31	802 ± 26	786 ± 26
		Accuracy [%]	Low MVPA	91.6 ± 0.9	89.2 ± 1.8	89.5 ± 1.6	91.0 ± 1.2	88.1 ± 1.9	88.3 ± 1.8
			High MVPA	92.2 ± 0.9	86.4 ± 1.9	85.1 ± 2.4	89.0 ± 2.3	90.7 ± 1.4	85.4 ± 2.1
Visual search	Simple	Response time [ms]	Low MVPA	595 ± 11	602 ± 10	581 ± 7	598 ± 13	597 ± 12	606 ± 12
			High MVPA	585 ± 11	568 ± 7	601 ± 12	576 ± 9	591 ± 12	580 ± 11
		Accuracy [%]	Low MVPA	89.0 ± 3.6	90.0 ± 3.1	88.3 ± 3.4	92.3 ± 2.8	94.3 ± 2.2	92.1 ± 3.2
			High MVPA	88.9 ± 3.4	92.6 ± 1.8	90.4 ± 2.7	94.4 ± 2.6	95.3 ± 2.3	87.9 ± 4.0
	Complex	Response time [ms]	Low MVPA	1,653 ± 69	1,657 ± 60	1,560 ± 56	1,666 ± 53	1,533 ± 62	1,616 ± 64
			High MVPA	1,613 ± 66	1,525 ± 61	1,537 ± 66	1753 ± 58	1,604 ± 56	1,589 ± 67
		Accuracy [%]	Low MVPA	91.4 ± 2.0	90.3 ± 2.3	92.4 ± 1.4	92.0 ± 2.4	91.8 ± 2.4	87.2 ± 3.8
			High MVPA	94.2 ± 1.6	93.4 ± 1.8	92.5 ± 1.7	97.4 ± 0.6	93.6 ± 2.9	95.4 ± 1.4

Data are mean ± SEM.

### Stroop test

#### Response times

Response times on the simple level of the Stroop test were similar between the PE and academic lesson trials (main effect of trial, *p* = 0.811), but did get quicker across the morning (main effect of time, *F*_(2,148)_ = 23.79, *p* < 0.001, partial η^2^ = 0.077). Additionally, response times on the simple level of the Stroop test were quicker overall in high-fit adolescents (main effect of fitness, *F*_(1,74)_ = 141.73, *p* < 0.001, partial η^2^ = 0.067); and in those who spent less time in MVPA during the PE lesson (main effect of MVPA, *F*_(1,69)_ = 40.70, *p* < 0.001, partial η^2^ = 0.021). However, the pattern of change in response times was similar between trials (trial * time, *p* = 0.823); and the pattern of change was not affected by fitness (trial * time * fitness, *p* = 0.476) or MVPA time (trial * time * MVPA, *p* = 0.416).

Overall, response times on the complex level of the Stroop test were quicker on the academic lesson trial than on the PE lesson trial (main effect of trial, *F*_(1,74)_ = 38.59.4, *p* < 0.001, partial η^2^ = 0.008) and got quicker across the morning (main effect of time, *F*_(2,148)_ = 33.39, *p* < 0.001, partial η^2^ = 0.078). Furthermore, response times on the complex level of the Stroop test were quicker overall in high-fit adolescents when compared to low-fit adolescents (main effect of fitness, *F*_(1,74)_ = 294.11, *p* < 0.001, partial η^2^ = 0.074); and in those who spent more time during the PE lesson in MVPA compared to those who spent less time in MVPA (main effect of MVPA, *F*_(1,69)_ = 106.52, *p* < 0.001, partial η^2^ = 0.034). However, the pattern of change in response times was similar between the academic lesson and PE lesson trials (trial * time, *p* = 0.232); and was not affected by fitness (trial * time * fitness, *p* = 0.933) or the time spent in MVPA (trial * time * MVPA, *p* = 0.128).

#### Accuracy

Accuracy on the simple level of the Stroop test was similar between trials (main effect of trial, *p* = 0.691); however, accuracy did improve across the morning (main effect of time, *F*_(2,148)_ = 10.13, *p* < 0.001, partial η^2^ = 0.119). Additionally, accuracy for the simple level of the Stroop test was not affected by fitness (main effect of fitness, *p* = 0.849) or MVPA (main effect of MVPA, *p* = 0.946). The pattern of change in accuracy was similar between the academic lesson trial and the PE lesson trial (trial * time, *p* = 0.129); and it was not affected by time spent in MVPA during the PE lesson (trial * time * MVPA, *p* = 0.483) or fitness (trial * time * fitness, *p* = 0.269).

Overall, accuracy on the complex level of the Stroop test was similar between trials (main effect of trial, *p* = 0.120) and did not change across the morning (main effect of time, *p* = 0.351). Additionally, accuracy for the complex level of the Stroop test was not affected by fitness (main effect of fitness, *p* = 0.220) or MVPA time during the PE lesson (main effect of MVPA, *p* = 0.150). Furthermore, the pattern of change in accuracy was similar between trials (trial * time, *p* = 0.987); and it was not affected by MVPA (trial * time * MVPA, *p* = 0.946) or fitness (trial * time * fitness, *p* = 0.746).

### Sternberg paradigm

#### Response times

**Figure 2:**
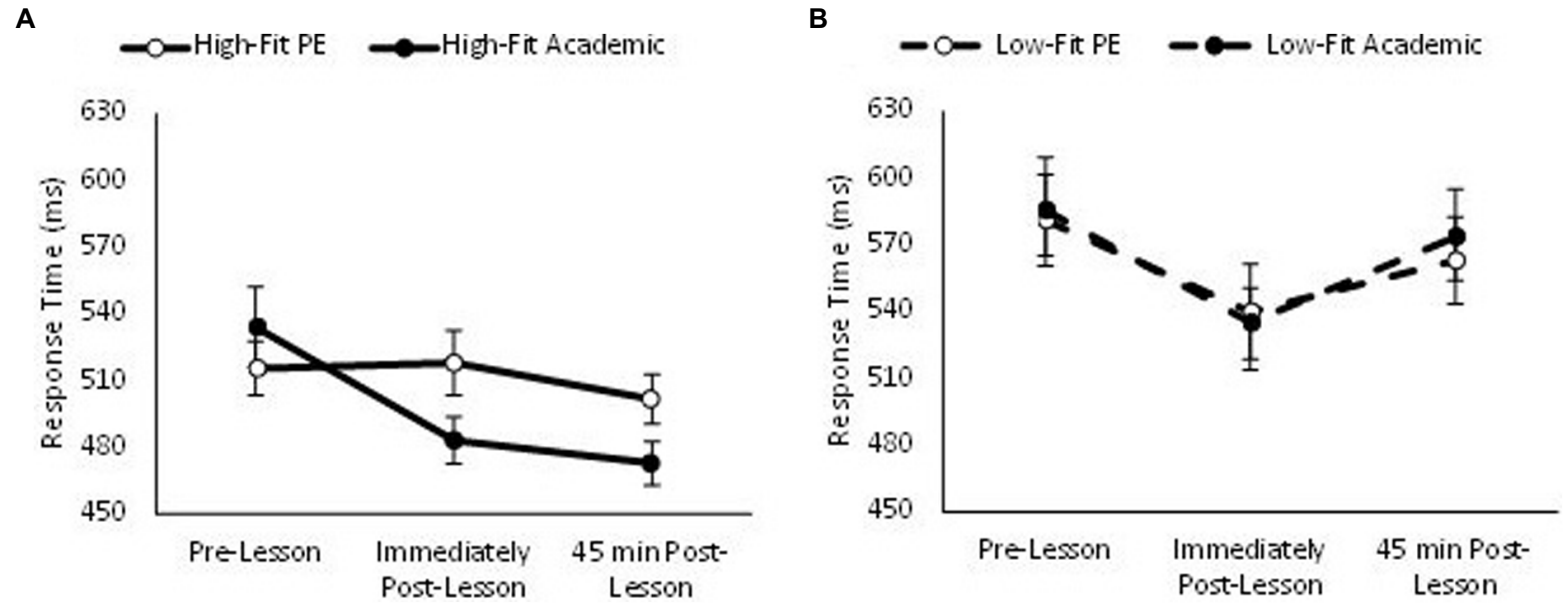
Response times across the morning on the one item level of the Sternberg paradigm on the PE and academic lesson trials for the high-fit (trial * time, *p* = 0.001; **A**) and low-fit (trial * time, *p* = 0.746; **B**) groups (trial * time * fitness, *p* = 0.021).

Overall, response times on the one item level of the Sternberg paradigm were quicker in the academic lesson trial than PE trial (main effect of trial, *F*_(1,74)_ = 4.20, *p* = 0.04, partial η^2^ = 0.006) and got quicker across the morning (main effect of time, *F*_(2,148)_ = 19.45, *p* < 0.001, partial η^2^ = 0.122). Additionally, response times on the one item level of the Sternberg paradigm were quicker overall in high-fit adolescents (main effect of fitness, *F*_(1,74)_ = 175.84, *p* < 0.001, partial η^2^ = 0.121); and in those who spent less time in MVPA compared to those who spent more time in MVPA during the PE lesson (main effect of MVPA, *F*_(1,69)_ = 73.11, *p* < 0.001, partial η^2^ = 0.052). The pattern of change in response times was similar between trials (trial * time, *p* = 0.114) and not affected by MVPA (trial * time * MVPA, *p* = 0.191). However, there was a trial * time * fitness interaction (*F*_(2,148)_ = 3.88, *p* = 0.021, partial η^2^ = 0.017; Figure 2). Upon further inspection, in high fit adolescents the improvement in response times across the morning was greater on the academic trial (trial * time, *F*_(2,72)_ = 6.80, *p* = 0.001, partial η^2^ = 0.089; Figure 2A); whilst in low fit adolescents the pattern of change in response times was similar between the PE and academic trial (trial * time, *p* = 0.746).

**Figure 3:**
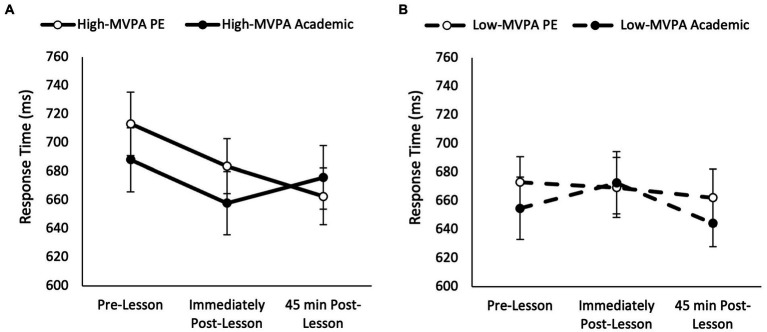
Response times across the morning on the three item level of the Sternberg paradigm on the PE and academic lesson trials for the high MVPA (trial * time, *p* = 0.012; **A**) and low MVPA (trial * time, *p* = 0.443; **B**) groups (trial * time * MVPA, *p* = 0.045).

Response times on the three item level of the Sternberg paradigm were quicker in the academic lesson trial than PE trial (main effect of trial, *F*_(1,74)_ = 13.07, *p* = 0.001, partial η^2^ = 0.041) and got quicker across the morning (main effect of time, *F*_(2,148)_ = 10.13, *p* < 0.001, partial η^2^ = 0.044). Response times on the three item level of the Sternberg paradigm were also quicker overall in high-fit adolescents when compared to low-fit adolescents (main effect of fitness, *F*_(1,74)_ = 260.28, *p* < 0.001, partial η^2^ = 0.100), and were influenced by MVPA, with high MVPA adolescents demonstrating a greater reduction in response times when compared with low MVPA adolescents (main effect of MVPA, *F*_(1,69)_ = 31.28, *p* < 0.001 partial η^2^ = 0.012). Furthermore, response time improved immediately post-lesson in both trials, with a tendency for further improvement 45 min post-lesson in the PE trial, this did not reach statistical significance (trial * time, *F*_(2,148)_ = 2.97, *p* = 0.052). Additionally, the pattern of change in response times was not affected by fitness (trial * time * fitness, *p* = 0.745). However, the pattern of change in response time across the morning was affected by the amount of MVPA completed in the PE lesson (time * trial * MVPA, *F*_(2,138)_ = 3.10, *p* = 0.045, partial η^2^ = 0.025; Figure 3). Upon further inspection, whilst response times improved across the morning on the PE trial in those who completed more MVPA during the PE lesson (trial * time, *F*_(2,68)_ = 4.41, *p* = 0.012, partial η^2^ = 0.063; Figure 3A), the pattern of change in response times across the morning was similar between the PE and academic trials for those who completed less MVPA (trial * time, *p* = 0.443; Figure 3B).

**Figure 4:**
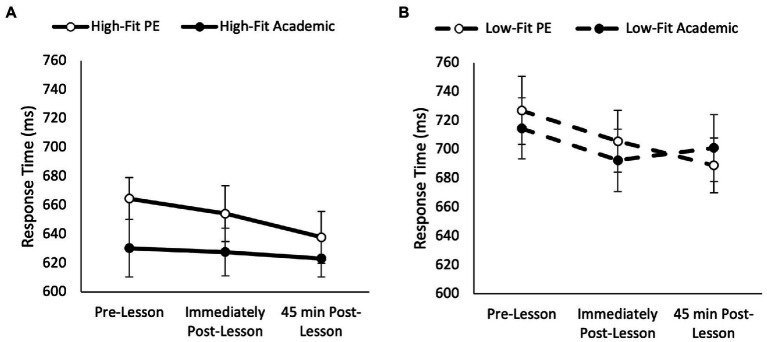
Response times across the morning on the five item level of the Sternberg paradigm on the PE and academic lesson trials for the high-fit (trial * time, *p* = 0.001; **A**) and low-fit (trial * time, *p* = 0.863; **B**) groups (trial * time * fitness, *p* = 0.023).

Overall, response times on the five item level of the Sternberg paradigm were similar between the academic lesson trial and the PE lesson trial (main effect of trial, *p* = 0.693), but response times got quicker across the morning (main effect of time, *F*_(2, 148)_ = 34.98, *p* < 0.001, partial η^2^ = 0.157). Additionally, response times on the five item level were quicker overall in high-fit adolescents when compared to low-fit adolescents (main effect of fitness, *F*_(1,74)_ = 26.15, *p* < 0.001, partial η^2^ = 0.014); and were influenced by MVPA time during the PE lesson (main effect of MVPA, *F*_(1,69)_ = 12.76, *p* < 0.001, partial η^2^ = 0.005) as high MVPA adolescents had quicker response time overall. Whilst the pattern of change in response times was similar between the academic lesson and PE lesson trials (trial * time, *p* = 0.071), and it was not affected by MVPA (trial * time * MVPA, *p* = 0.203); the pattern of change in response times was significantly affected by fitness (trial * time * fitness, *F*_(2,148)_ = 3.76, *p* = 0.023, partial η^2^ = 0.028; Figure 4). Upon further inspection, response times improved immediately and 45 min following the PE lesson in high fit adolescents when compared to the academic trial (trial * time, *F*_(2,72)_ = 6.59, *p* = 0.001, partial η^2^ = 0.093; Figure 4A). However, there was no difference in the change in response times between the PE and academic trials in the low fit adolescents (trial * time, *p* = 0.863).

#### Accuracy

Accuracy on the one item level of the Sternberg paradigm was similar between the PE and academic trials (main effect of trial, *p* = 0.839), but did improve across the morning (main effect of time, *F*_(2,148)_ = 6.86, *p* = 0.011, partial η^2^ = 0.002). In addition, accuracy on the one item level of the Sternberg paradigm was not different between low and high-fit adolescents (main effect of fitness, *p* = 0.297). Neither was accuracy affected by MVPA when comparing those adolescents with a high MVPA against those with low MVPA in the PE lesson (main effect of MVPA, *p* = 0.774). The pattern of change was similar between trials (trial * time, *p* = 0.659) and was not affected by fitness (time * trial * fitness, *p* = 0.453) or MVPA during the PE lesson (trial * time * fitness, *p* = 0.312).

Overall, on the three item level of the Sternberg paradigm, accuracy was similar between trials (main effect of trial, *p* = 0.850) and did not change across the morning (main effect of time, *p* = 0.987). Additionally, when comparing high and low-fit adolescents, accuracy was not different (main effect of fitness, *p* = 0.767), nor was it different when comparing adolescents with high and low MVPA during the PE lesson (main effect of MVPA, *p* = 0.344). The pattern of change for accuracy was similar between trials (trial * time, *p* = 0.633) and the pattern of change was not affected by fitness (time * trial * fitness, *p* = 0.807) or MVPA time during the PE lesson (time * trial * MVPA, *p* = 0.206).

**Figure 5:**
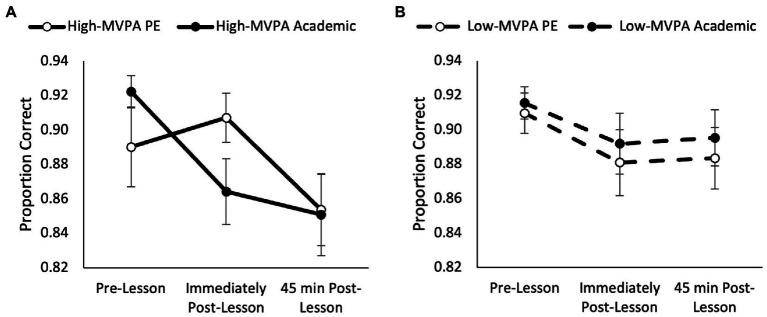
Accuracy presented as the proportion of correct responses across the morning on the five item level of the Sternberg paradigm for the high MVPA (trial * time, *p* = 0.023; **A**) and low MVPA (trial * time, *p* = 0.946; **B**) groups (trial * time * MVPA, *p* = 0.036).

For the five item level of the Sternberg paradigm, accuracy was similar across trials (main effect of trial, *p* = 0.669), but accuracy was reduced across the morning (main effect of time, *F*_(2,148)_ = 9.99, *p* < 0.001, partial η^2^ = 0.117). Accuracy on the five item level of the Sternberg paradigm was not affected by fitness (main effect of fitness, *p* = 0.175) or by MVPA (main effect of MVPA, *p* = 0.407). Whilst the pattern of change for accuracy was similar between trials (trial * time, *p* = 0.128), and it was not affected by fitness (trial * time * fitness, *p* = 0.805); it was affected by MVPA during the PE lesson (trial * time * MVPA, *F*_(2,138)_ = 3.41, *p* = 0.036, partial η^2^ = 0.047; Figure 5). Upon further inspection, accuracy improved immediately following the PE lesson in those who completed more MVPA during the PE lesson when compared to the academic lesson (trial * time, *F*_(2,68)_ = 4.30, *p* = 0.023, partial η^2^ = 0.119; Figure 5A). However, there was no different in accuracy between the PE and academic trials in those who completed less MVPA during the PE lesson (trial * time, *p* = 0.946; Figure 5B).

### Visual search

#### Response times

**Figure 6:**
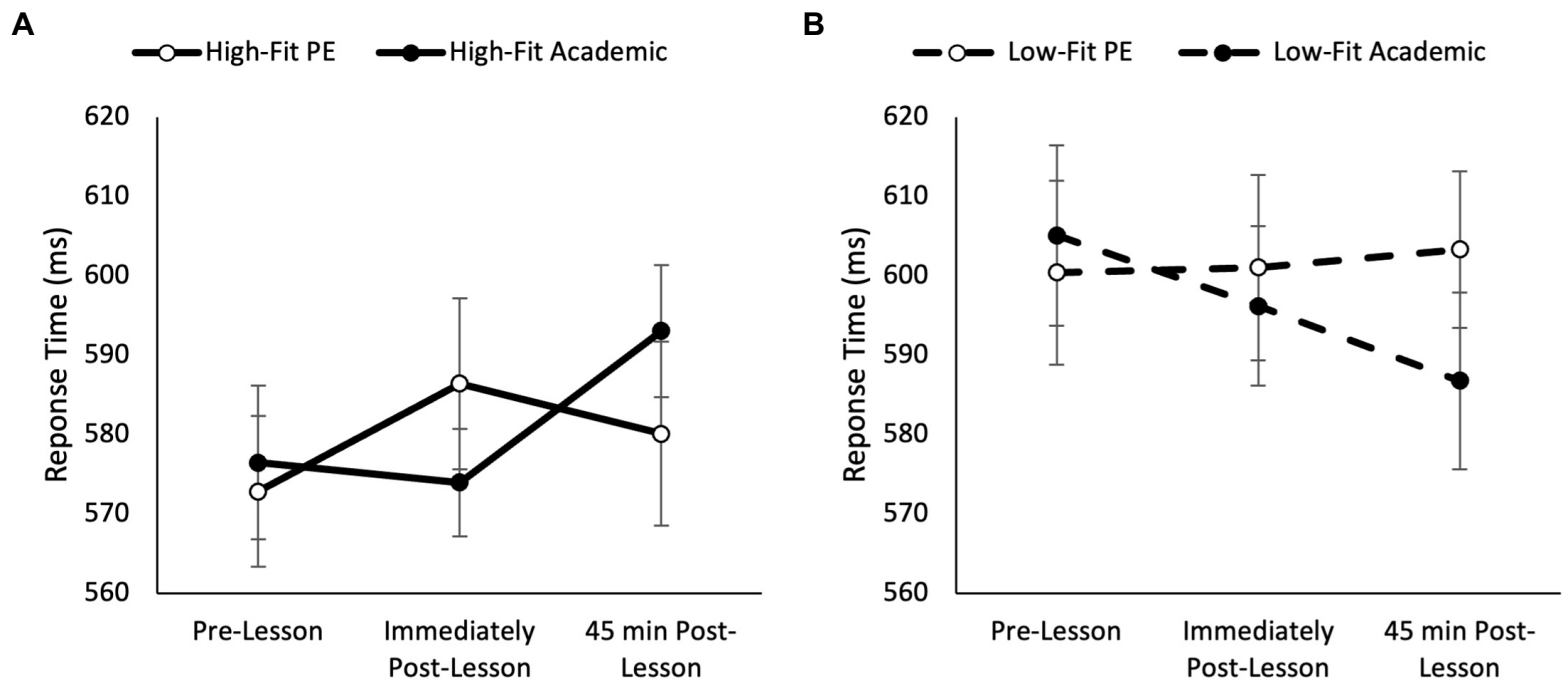
Response times across the morning on the simple level of the visual search on the PE and academic lesson trials for the high-fit (trial * time, *p* = 0.036; **A**) and low-fit (trial * time, *p* = 0.130; **B**) groups (trial * time * fitness, *p* = 0.018).

Overall, response times on the simple level of visual search were similar for the academic lesson and PE lesson trials (main effect of trial, *p* = 0.534) and did not improve across the morning (main effect of time, *p* = 0.822). Response times on the simple level of the visual search were quicker overall in high-fit adolescents when compared to low-fit adolescents (main effect of fitness, *F*_(1,74)_ = 34.74, *p* < 0.001, partial η^2^ = 0.043); and quicker in those who spent more time during the PE lesson in MVPA compared to those who spent less time in MVPA (main effect of MVPA, *F*_(1,69)_ = 16.69, *p* < 0.001, partial η^2^ = 0.022). Whilst the pattern of change in response times was similar between the academic lesson and PE lesson trials (trial * time, *p* = 0.305), the pattern of change in response times was affected by fitness (trial * time * fitness, *F*_(2,148)_ = 4.02, *p* = 0.018, partial η^2^ = 0.021; Figure 6). Upon further inspection, in high fit adolescents response times were slower immediately following the PE lesson and 45 min following the academic lesson (trial * time, *F*_(2,72)_ = 3.34, *p* = 0.036, partial η^2^ = 0.049; Figure 6A); whilst the pattern of change in response times was similar following the PE and academic lesson in low fit adolescents (trial * time, *p* = 0.130; Figure 6B).

**Figure 7:**
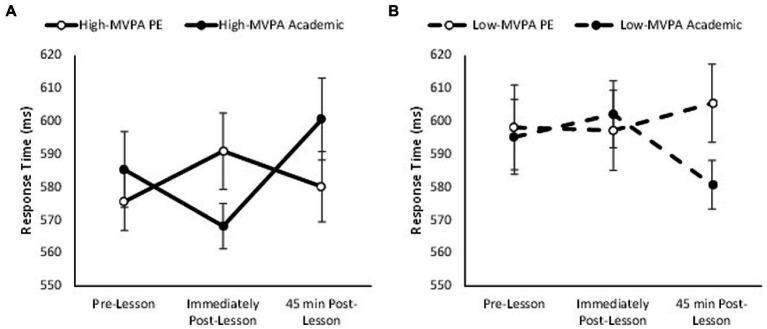
Response times across the morning on the simple level of the visual search on the PE and academic lesson trials for the high MVPA (trial * time, *p* < 0.001; **A**) and low MVPA (trial * time, *p* = 0.026; **B**) groups (trial * time * MVPA, *p* < 0.001).

Additionally, response times on the simple level were also affected by the amount of MVPA completed during the PE lesson (trial * time * MVPA, *F*_(2,138)_ = 10.18, *p* < 0.001, partial η^2^ = 0.071; Figure 7). Upon further inspection, in those who completed more MVPA response times were slower immediately following the PE lesson and 45 min following the academic lesson (trial * time, *F*_(2, 68)_ = 7.53, *p* < 0.001, partial η^2^ = 0.117; Figure 7A); whilst in those who completed less MVPA, response times were maintained across the morning on the PE trial, and improved 45 min following the academic lesson (trial * time, *F*_(2,72)_ = 3.64, *p* = 0.026, partial η^2^ = 0.046; Figure 7B).

**Figure 8:**
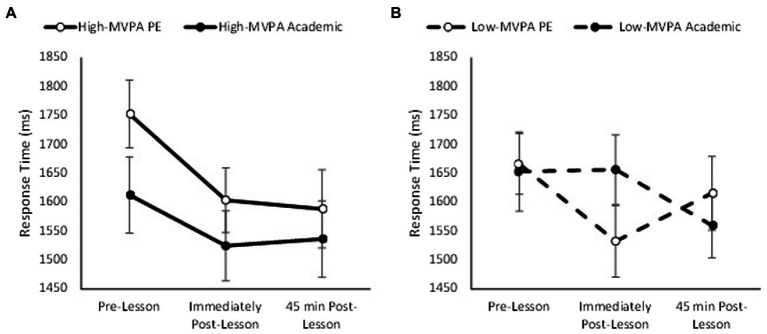
Response times across the morning on the complex level of the visual search on the PE and academic lesson trials for the high MVPA (trial * time, *p* = 0.265; **A**) and low MVPA (trial * time, *p* = 0.003; **B**) groups (trial * time * MVPA, *p* = 0.037).

Overall, response times on the complex level of the visual search test were quicker on the academic lesson trial than in the PE lesson trial (main effect of trial, *F*_(1,69)_ *=* 7.97, *p* = 0.005, partial η^2^ = 0.024) and improved across the morning (main effect of time, *F*_(2,138)_ = 16.39, *p* < 0.001, partial η^2^ = 0.115). Response times on the complex level of the visual search test were not affected by fitness (main effect of fitness, *p* = 0.147) or MVPA time during the PE lesson (main effect of MVPA, *p* = 0.616). Whilst the pattern of change in response times was similar between the academic lesson and PE lesson trials (trial * time, *p* = 0.062), and was not affected by fitness (trial * time * fitness, *p* = 0.334); the pattern of change was affected by MVPA (trial * time * MVPA, *F*_(2,138)_ = 3.31, *p* = 0.037, partial η^2^ = 0.030; Figure 8). Specifically, the pattern of change in response times was similar across the PE and academic trials in adolescents who completed more MVPA during the PE lesson (trial * time, *p* = 0.265; Figure 8A). However, in those adolescents who completed less MVPA, response times improved immediately following the PE lesson (trial * time, *F*_(2,72)_ = 5.72, *p* = 0.003, partial η^2^ = 0.093; Figure 8B).

#### Accuracy

Accuracy on the simple level of the visual search was similar between the PE and academic lesson trials (main effect of trial, *p* = 0.212), and did not change across the morning (main effect of time, *p* = 0.525). Additionally, when comparing high-fit to low-fit adolescents, accuracy was not affected by fitness (main effect of fitness, *p* = 0.681). Likewise, when comparing those adolescents with high MVPA during the PE lesson against those with low MVPA, accuracy was similar (main effect of MVPA, *p* = 0.812). The pattern of change was similar between trials (time * trial, *p* = 0.701) and the pattern of change between trials was not affected by fitness (time * trial * fitness, *p* = 0.405) or by MPVA during the PE lesson (time * trial * MVPA, *p* = 0.415).

Overall, accuracy on the complex level of the visual search was similar between the PE and academic lesson trials (main effect of trial, *p* = 0.665), and did not change across the morning (main effect of time, *p* = 0.475). Furthermore, when comparing high-fit to low-fit adolescents, accuracy was not affected (main effect of fitness, *p* = 0.358). However, adolescents who completed more MVPA in the PE lesson had greater overall accuracy than those who completed less MVPA (main effect of MVPA, *F*_(1,69)_ = 3.96, *p* = 0.050, partial η^2^ = 0.054). The pattern of change was similar between trials (time * trial, *p* = 0.355) and the pattern of change between trials was not affected by fitness (time * trial * fitness, *p* = 0.358) or by MPVA during the PE lesson (time * trial * fitness, *p* = 0.227).

## Discussion

The main findings of the present study are that a 60 min games-based PE lesson had no effect on perception, working memory, attention, or executive function in adolescents, when compared to a standard academic lesson. However, adolescents who spent a higher percentage of their PE lesson undertaking MVPA experienced some cognitive benefits, as evidenced by improvements in the speed (three item level) and accuracy (five item level) of working memory (as assessed by the Sternberg paradigm). Additionally, the cognitive response to the PE lesson was influenced by fitness as high-fit adolescents demonstrated improvements in speed of working memory, as evidenced by improved response times on the five-item level of the Sternberg paradigm. Furthermore, the present study also shows that high fit adolescents display superior cognition when compared to their lower fit counterparts, across all domains of cognitive function. Finally, MVPA time in the single-gender games-based PE lessons observed in this study was greater than that previously reported for adolescents. On average, adolescents spent 67% of the lesson time undertaking MVPA; although considerable inter-individual variation was evident as time spent in MVPA ranged from 23% in the least active, to 90% in the most active, adolescents.

The present study is the first to examine the acute cognitive response to a games-based PE lesson in adolescents. Overall, perception, working memory, and attention were unchanged following a games-based PE lesson. These results are consistent with those previously reported by Williams et al. (2020), whereby, overall, cognitive function remained unchanged in adolescents following a 60 min football session. The unaffected cognitive response to the 60 min games-based activity reported in this study, and by Williams et al. (2020), could be attributed to the duration of the exercise protocol. Haverkamp et al. (2020) recently reported a larger effect on cognitive function when the acute exercise interventions are of a shorter duration. However, Cooper et al. (2018) previously reported enhanced working memory and executive function in adolescents following a 60 min games-based activity (basketball). The inconsistent findings could be attributed to the activity patters of the respective exercise protocols, as Cooper et al. (2018) reported higher average heart rate than that reported by Williams et al. (2020) and the current study. The pedagogical requirement of a PE lesson could have attributed to a less intense exercise protocol, and resultantly, a lower average heart rate in the current study. Future work should investigate the acute cognitive response to games-based activity of varying intensity and duration.

The present study also demonstrated that high fit adolescents displayed superior cognition compared to their lower fit counterparts, with high fit adolescents exhibiting quicker response times for all domains of cognitive function assessed. The effect of fitness on perception (visual search test) is consistent with the findings of Williams et al. (2022), whereby, the beneficial effect of fitness on visual processing speed was first demonstrated. High fit adolescents also demonstrated quicker response times for attention and working memory, which is consistent with recent evidence of the enhancing effect of fitness on these domains of cognitive function (Cooper et al., 2016; Aadland et al., 2017; Haverkamp et al., 2020; Williams et al., 2020). It has previously been stated that participation in exercise modifies the capacity of the nervous system to adapt its organisation to altered demands and environment, termed neuroplasticity (Hötting and Röder, 2013). Engaging in repetitive aerobic physical activity induces increases in angiogenesis (Best, 2010) and the availability of certain neurotrophins, especially brain-derived neurotrophic factor (BDNF; Cho et al., 2012), which are prerequisites for neuroplasticity (Hötting and Röder, 2013). Consequently, increased angiogenesis and BDNF have been suggested as an explanation for the beneficial effect of fitness on cognitive function (Haverkamp et al., 2020). However, it has recently been demonstrated that there is no association between fitness and BDNF concentration in young people (Williams et al., 2022). Therefore, whilst the current study supports the beneficial effect of fitness on cognitive function, future work should seek to explore the potential mechanisms for this association.

The acute cognitive response to exercise was influenced by the participant’s fitness in the present study, with high-fit adolescents demonstrating greater improvements in working memory following the PE lesson. This finding is consistent with the previous evidence that cognitive function is enhanced to a greater extent in high-fit adolescents following an acute bout of games-based activity, when compared to low-fit adolescents (Cooper et al., 2018; Williams et al., 2020). However, in the present study, high-fit adolescents only demonstrated improved working memory following their PE lesson, whereas Cooper et al. (2018) were also able to evidence improved attention and executive function in high-fit adolescents following an acute bout of games-based activity. As the acute exercise-cognition relationship is influenced by moderating factors (e.g., age, physical fitness) and exercise characteristics (e.g., duration, intensity) (Williams et al., 2019), a potential explanation for the discrepant findings could be the higher exercise intensity reported by Cooper et al. (2018) than in the present study. Furthermore, the enhanced cognitive function reported by Williams et al. (2020) in low-fit adolescents when not undertaking exercise, was not reflected in the current study. A potential explanation for not replicating these findings could be that on average, overall fitness was higher for adolescents in the present study when compared with the overall average fitness reported by Williams et al. (2020); and, the difference between fitness for the low and high fit groups was smaller for the present study than previously reported (Williams et al., 2020). The current study presents the novel finding that games-based PE lessons improve cognitive function in high-fit adolescent. Also highlighting that the exercise protocol and individual participant characteristics influence the cognitive response and should be considered in future research.

Supporting the concept that the intensity of physical activity is important for the subsequent cognitive effects, a novel finding of the present study is that the acute cognitive response to a games-based PE lesson is enhanced for those adolescents who spent more time undertaking MVPA during their PE lesson. Specifically, adolescents with high MVPA during the PE lesson demonstrated improved working memory immediately post-PE lesson; working memory was also enhanced 45-min post-PE lesson. The absence of collinearity between fitness and MVPA amongst participants in the present study, when combined with the positive effect of MVPA on working memory, suggests MVPA enhances acute cognitive function, independent of fitness. An explanation for these findings could be the potential influence of physical activity intensity on functional connectivity across brain regions, primarily those involved in memory and executive function (Moore et al., 2021); resultantly, the efficiency of evaluating the stimulus is increased (Chang et al., 2013). The findings from previous studies investigating the role of physical activity intensity on cognitive function are equivocal, with positive (Syväoja et al., 2013; Lee et al., 2014), inconsistent (Aadland et al., 2017), and negative (Cadenas-Sanchez et al., 2020; Ludyga et al., 2020; Williams et al., 2022) results. The discrepancy between our findings and those previously reported could be attributed to methodological differences, such as utilising self-report measures of physical activity (Syväoja et al., 2013; Aadland et al., 2017); or, the assessment of habitual physical activity intensity (Lee et al., 2014; Cadenas-Sanchez et al., 2020; Williams et al., 2022). Whereas, due to the dynamic relationship between exercise and circulating neurotrophic factors that enhance cognitive function (Cho et al., 2012), the present study investigated the impact of physical activity intensity on cognitive function immediately following a bout of physical activity. Therefore, to the authors’ knowledge, our study is the first to examine the effect of device-measured intensity during a single bout of physical activity on acute cognitive function in adolescents, demonstrating that physical activity behaviour impacts working memory in adolescents.

The present study provides valuable insight into the activity patterns during 60 min single-gender, games-based PE lessons. Overall, across the four lessons observed, MVPA was undertaken for 67 ± 14% (40 ± 8 min) of the timetabled lesson time (60 min). Therefore, the recommended minimum 50% of PE lesson time spent in MVPA was exceeded (Association for Physical Education, 2020). Interestingly, MVPA time did not differ between high and low fit adolescents, suggesting that MVPA time during a PE lesson is independent of fitness. Whilst average MVPA time exceeded 50%, time spent in MVPA ranged from 23% in the least active to 90% in the most active adolescents. Consequently, the opportunity for PE to allow all students meet the 60 min of MVPA per day recommendation was not maximised and future work should explore how PE can be modified to increase the amount of MVPA time for the least active students. The MVPA time reported in the present study is higher than the 48.6% (41.3–55.9%) of lesson time previously reported for adolescent PE lessons by Hollis et al. (2017) in their systematic review and meta-analysis. It has previously been stated that MVPA time varies according to the type of activity students engaged in, with team invasion game lessons (e.g., basketball and football) eliciting higher MVPA time than dance, gymnastics, or individual direct competition lessons (Fairclough and Stratton, 2005). However, the MVPA time in the present study is higher than that reported by Fairclough and Stratton (2005) for team invasion game lessons (46%). The higher MVPA reported in the present study could be a result of changes in PE over time and/or the recommendation by the Association for Physical Education (2020) to increase MVPA time in lesson; or, methodological inconsistencies, including the use of observational methods to monitor MVPA time in previous work (Fairclough and Stratton, 2005). Whilst the present study provides a novel contribution to the understanding of MVPA during PE lessons using device-based measures of activity, future studies should examine device measured MVPA in PE lessons across all domains of the national curriculum.

Whilst the present study provides novel insight regarding the effects of curriculum PE lessons on subsequent cognitive function in adolescents, it is not without limitations. Firstly, the present study only recruited participants from a single school year (UK year 8), and given the changes in physical activity and fitness that occur across adolescence, the influence of the PE lesson on cognitive function might be different across stages of adolescence. Additionally, as the present study observed football PE lessons at a single school, the generalisability is limited. Future work should observe PE lessons across several secondary schools to better reflect PE nationally. Moreover, future work across multiple secondary schools would permit further exploration of fitness and MVPA as continuous variables, rather than categorical variables as used in the present study. Furthermore, whilst a games-based PE lesson is a domain of the national curriculum for PE in the United Kingdom (team direct competition), the cognitive response to a PE lesson was not assessed across the remaining domains of the national curriculum; and it cannot be assumed that the responses to all types of PE would be the same. Therefore, future work should explore the influence of all domains of the national curriculum for PE on cognitive function, across all stages of adolescence. Likewise, the examination of MVPA time and exercise intensity was limited to a games-based activity lesson; MVPA and the intensity for all domains of PE lessons should be established in future works.

## Conclusion

In summary, the present study highlights that the acute cognitive responses to a games based-PE lesson are moderated by physical activity intensity, whereby adolescents who completed more MVPA during the PE lesson experienced greater cognitive benefits. The findings of the present study would suggest that, if physical activity intensity during PE lessons is high, those lessons will enhance subsequent cognition and ultimately contribute to enhancing academic achievement. Furthermore, the acute cognitive response to a games based-PE lesson is moderated by fitness, whereby high-fit adolescents experienced improved cognitive function compared to their lower-fit peers. Finally, the present study contributes to the growing body of evidence that high fit adolescents demonstrate superior cognition than their lower fit counterparts; highlighting the importance of interventions aimed at improving fitness in this population.

## Data availability statement

The raw data supporting the conclusions of this article will be made available by the authors, without undue reservation.

## Ethics statement

This study was approved by Nottingham Trent University School of Science and Technology Human Invasive Ethics Committee.

## Author contributions

LG, KD, RW, and SC contributed to the conception and design of the study. LG, KD, RW, RB, CS, JM, and SC contributed to data collection. LG and SC performed the statistical analysis. LG wrote the first draft of the manuscript. All authors contributed to the manuscript revision, read, and approved the submitted version.

## Conflict of interest

The authors declare that the research was conducted in the absence of any commercial or financial relationships that could be construed as a potential conflict of interest.

## Publisher’s note

All claims expressed in this article are solely those of the authors and do not necessarily represent those of their affiliated organizations, or those of the publisher, the editors and the reviewers. Any product that may be evaluated in this article, or claim that may be made by its manufacturer, is not guaranteed or endorsed by the publisher.
